# In the national epidemiological bulletins – a selection from current issues

**DOI:** 10.2807/1560-7917.ES.2016.21.31.30307

**Published:** 2016-08-04

**Authors:** Eurosurveillance editorial team

**Affiliations:** 1 European Centre for Disease Prevention and Control (ECDC), Stockholm, Sweden

**Keywords:** public health, Europe

## Healthcare-associated infections and antibiotic resistance

### Glycopeptides-resistant Enterococci in healthcare facilities in France: Epidemiological data from the healthcare-associated infection early warning and response system, July 2001 - June 2015

Bulletin épidémiologique hebdomadaire 24-25/2016

26 July 2016, France

http://invs.santepubliquefrance.fr/beh/2016/24-25/2016_24-25_3.html

### Surveillance of *Enterococcus* spp. causing bacteraemia in England, Wales and Northern Ireland

Health Protection Report; 10(23)

15 July 2016, United Kingdom

https://www.gov.uk/government/uploads/system/uploads/attachment_data/file/541028/hpr2316.pdf

## Sexually transmitted diseases

### Annual report from the sentinel surveillance study of blood borne virus testing in England: 2015

Health Protection Report; 10(24)

22 July 2016, United Kingdom

https://www.gov.uk/government/uploads/system/uploads/attachment_data/file/541032/hrp2416.pdf

### Unlinked anonymous HIV and viral hepatitis monitoring among people who inject drugs: 2016 report

Health Protection Report; 10(23)

15 July 2016, United Kingdom

https://www.gov.uk/government/uploads/system/uploads/attachment_data/file/541028/hpr2316.pdf

### Sexually transmitted infections and chlamydia screening in England, 2015

Health Protection Report; 10(22)

08 July 2016, United Kingdom

https://www.gov.uk/government/uploads/system/uploads/attachment_data/file/543433/hpr2216_crrctd2.pdf

### Updated advice on the prevention of sexually transmitted Zika virus infection

Folkehelseinstituttet website

20 July 2016

https://www.fhi.no/nyheter/2016/oppdaterte-rad-om-forebygging-av-zikavirus-infeksjon-etter-opphold-i-utbrud/

## Blood-borne diseases

### Transfusion transmitted infections (UK): 2015

Health Protection Report; 10(24)

22 July 2016, United Kingdom

https://www.gov.uk/government/uploads/system/uploads/attachment_data/file/541032/hrp2416.pdf

## Hepatitides

### Hepatitis C in 2015

Epidemiologisches Bulletin 29, 2016

25 July 2016, Germany

http://www.rki.de/DE/Content/Infekt/EpidBull/Archiv/2016/Ausgaben/29_16.pdf?__blob=publicationFile

### Laboratory reports of hepatitis A infection, and hepatitis C: 2015

Health Protection Report; 10(24)

22 July 2016, United Kingdom

https://www.gov.uk/government/uploads/system/uploads/attachment_data/file/541032/hrp2416.pdf

## Food- and waterborne diseases

### Food and waterborne infections in 2015

Folkehelseinstituttet website

07 July 2016

https://www.fhi.no/nyheter/2016/mat-og-vannbarne-infeksjoner/

## Vaccine-preventable diseases

### National measles outbreak – April to July 2016

Epi-Insight 2016;17(8)

August 2016, Ireland

http://ndsc.newsweaver.ie/epiinsight/xvz2wrpu8jr?a=1&p=50630336&t=17517774

### Increased occurrence of whooping cough 

EPI-NEWS 27a+b, 2016

6 July 2016, Denmark

http://www.ssi.dk/English/News/EPI-NEWS/2016/No%2027%20-%202016.aspx

### Tetanus death in unvaccinated pensioner - a case report from Bavaria

Epidemiologisches Bulletin 30, 2016

1 August 2016, Germany

http://www.rki.de/DE/Content/Infekt/EpidBull/Archiv/2016/Ausgaben/30_16.pdf?__blob=publicationFile

## Zoonoses

### Malaria imported into the UK: 2015

Health Protection Report; 10(24)

22 July 2016, United Kingdom

https://www.gov.uk/government/uploads/system/uploads/attachment_data/file/541032/hrp2416.pdf

